# Training competence questionnaire for endurance and strength training: development and validation

**DOI:** 10.3389/fspor.2026.1909973

**Published:** 2026-08-07

**Authors:** Johannes Große Siemer, Michael Braksiek, Tim Below, Gerd Thienes

**Affiliations:** Department of Sports Science, University of Vechta, Vechta, Germany

**Keywords:** endurance, questionnaire, strength, training, training competence, validation, training quality

## Abstract

**Introduction:**

Endurance and strength training is frequently self-managed, yet no validated instrument assesses the competence required to plan, execute, and monitor/adjust one's own training from an exercise-science perspective. The Training Competence Questionnaire for Endurance and Strength (TCQ-E/S) was therefore developed, and initial validity evidence was provided.

**Methods:**

Data from 1,039 young adults in Germany were used to validate the instrument. Using a split-sample design, exploratory factor analysis (*n* = 692) and confirmatory factor analysis (*n* = 347) were conducted, a second-order model was tested, and convergent and discriminant validity as well as measurement invariance across sex, current sport participation, and sports club membership were evaluated. Test-retest reliability was assessed over a two-week interval in an independent subsample (*n* = 99).

**Results:**

Exploratory factor analysis supported a six-factor structure spanning planning, execution, and monitoring/adjustment, specified separately for endurance and strength. Confirmatory factor analysis showed acceptable model fit [CFI = .912, TLI = .908, RMSEA = .054 (90% CI .051–.057), SRMR = .051] with substantial standardized loadings and supported a hierarchical model with two second-order factors (endurance and strength training competence). Convergent and discriminant validity were supported. Scalar invariance held across all grouping variables. Internal consistency was high, and test–retest ICCs indicated moderate-to-good temporal stability.

**Discussion/conclusion:**

The TCQ-E/S is a theoretically grounded, multidimensional self-report instrument with initial evidence of factorial validity, reliability, and subgroup invariance. It enables the assessment of individual training competence in endurance and strength training, thereby allowing competence profiles to be compared between subgroups and supporting longitudinal research. Practically, the instrument offers applied sport and exercise settings a means to detect competence gaps in self-directed exercisers, to tailor guidance to individual needs, and to evaluate interventions aimed at fostering training competence, thereby complementing motivation-focused approaches to promoting effective and sustainable training behavior.

## 1 Introduction

A large share of sport and exercise participation takes place outside organized sport club structures and is therefore mainly self-directed. For Germany, evidence suggests that most sport participation occurs in informal, self-organized settings, with fitness and running/jogging among the most commonly practiced activities and typically pursued without formal coaching (1). Comparable patterns have been reported across Europe and North America, where running and fitness-related activities regularly appear among the most popular adult activities (2) and participation in commercial fitness facilities is widespread (3). From a public health perspective, both endurance-oriented activity and muscle-strengthening exercise are central components of contemporary physical activity recommendations, as they contribute to cardiometabolic health, musculoskeletal function, and overall well-being (4). Beyond health, endurance capacity and muscular strength are widely recognized as key performance determinants across many sports, and resistance training is increasingly viewed as a central training modality for enhancing sport-specific performance and reducing injury risk (5). Accordingly, recreational exercisers and athletes alike often train with goals that extend beyond “being active” *per se* and include mastery and performance related motives, like improving fitness, running faster, or lifting heavier weights (6).

However, meaningful progress depends on more than simply exercising: adaptations hinge on training quality, both at the level of the longer-term training process (e.g., goal setting, systematic progression, and the application of training principles) and at the level of the individual session (7). Creating such a coherent process is knowledge- and skill-intensive, requiring the integration of evidence-based principles, individual characteristics, and contextual constraints (8). Importantly, the responsibility for making these decisions often lies with the individual: In informal recreational settings, structured supervision is frequently absent and implementing structured training is often considered difficult (9). Consistent with this, evidence suggests that even regular exercisers possess only modest training-related knowledge (10). Even in competitive sport, athletes may train in decentralized models where day-to-day supervision by coaches or support staff is limited (11). Insufficient competence in managing training decisions may therefore contribute to inefficient progress and, in some cases, maladaptive load exposure or injury risk.

To compensate, exercisers increasingly turn to social media, online video platforms, and—more recently—generative artificial intelligence tools. Online fitness content is widely used by young adults (12), but its practical value is often constrained as training related information is generic, insufficiently individualized, or not grounded in exercise science (13). Similar concerns have been raised for AI-generated training plans, which often require expert review to ensure appropriate progression and individualization. Crucially, AI-assisted guidance does not eliminate the need for user competence; rather, it shifts it: the quality of the output depends on how precisely users can describe their goals, constraints, and training status, and on whether they can judge and implement recommendations appropriately (14).

Despite its practical relevance, no validated instrument currently captures individuals’ competence to self-manage their own training process (i.e., to plan, execute, and monitor/adjust training over time) from a training- and exercise-science perspective. Although such competence would ideally be assessed objectively, through demonstrated performance, this is resource-intensive and difficult to realize at scale; capturing perceived competence through self-report is therefore an established and widely used approach in competence research, as it is economical and applicable in large samples (15). Beyond the absence of a suitable instrument, training quality has been identified as an underexplored topic in sports science, alongside explicit calls for more research on how training quality can be assessed and improved (8). Available instruments address adjacent but different constructs. For example, control competence within the physical activity-related health competence (PAHCO) framework was developed primarily for health-enhancing physical activity (16) and does not differentiate training-specific process components such as explicit goal setting, structured exercise prescription, systematic monitoring of training effects, and longitudinal adjustment. Other instruments and concepts either assess perceived session quality (17) or focus on motor skill proficiency and foundational movement patterns in resistance training (18, 19), but they do not operationalize competence as an integrated capacity to manage one's training process across sessions and over time. Against this backdrop, the following section introduces a model of individual training competence as the conceptual basis for the instrument developed in this study.

### 1.1 The model of training competence

**Figure 1:**
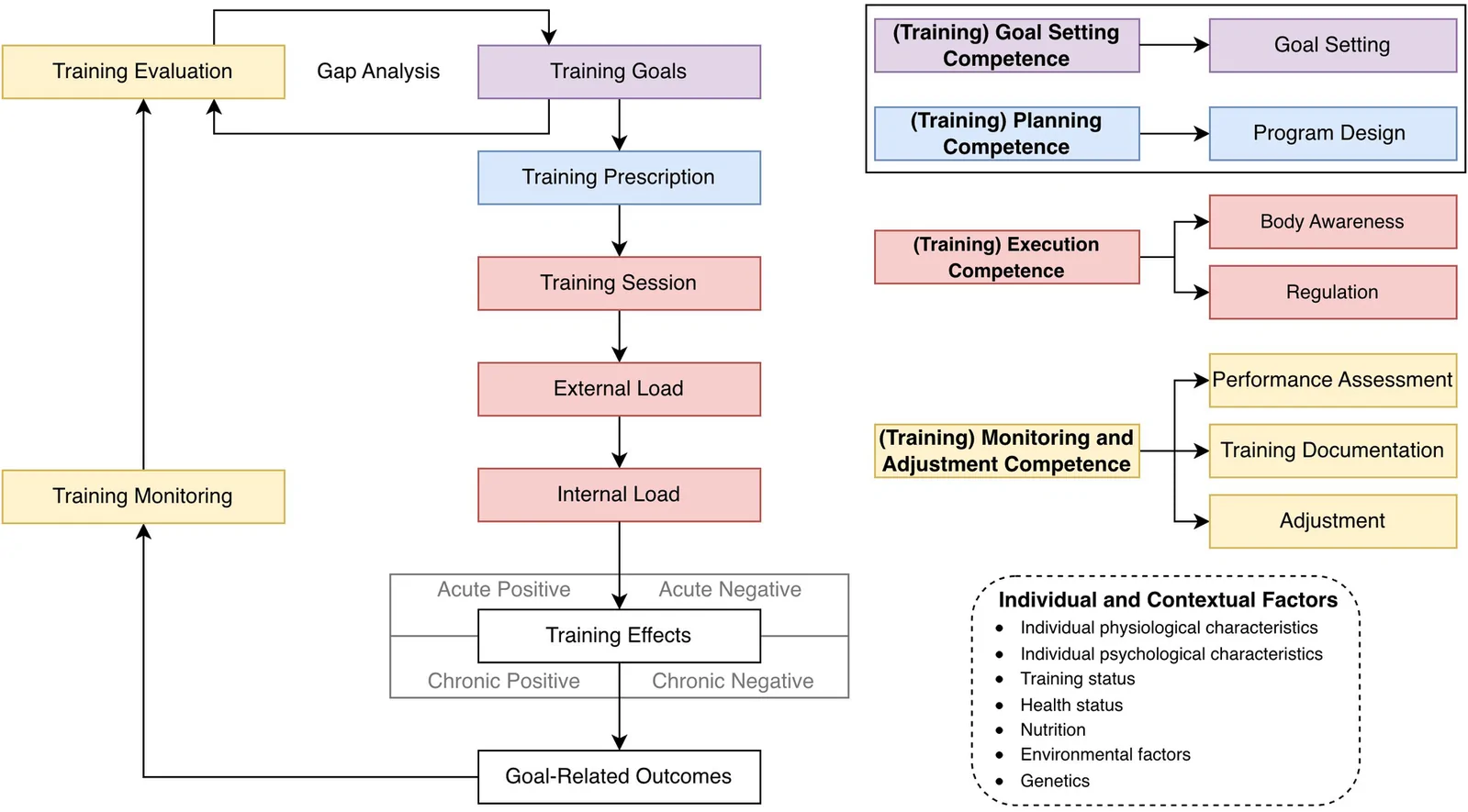
Conceptual model of individual training competence embedded in the training process [based on Jeffries et al. (25) and Impellizzeri et al, (27)]. The figure illustrates the theoretically proposed structure of training competence. In the empirical model, goal-setting competence was not retained as a distinct factor, as the corresponding items loaded on the training planning competence factor. The black frame therefore indicates that goal-setting competence and training planning competence were empirically combined into a single training planning competence factor.

Sport and exercise science lacks an established way to define and assess the competencies individuals need to independently plan, execute, and monitor/adjust their own training at a certain quality. To address this gap, a model of individual training competence is proposed (see Figure 1). To anchor the proposed model in an established competence tradition, it draws on the conceptual logic of physical activity-related health competence (16) and transfers its underlying understanding of competence to the training and exercise context. In this functional-pragmatic perspective from educational research, competencies are learnable, context- and demand-specific dispositions that integrate “to know” (knowledge), “to be able” (skills and abilities, including physical-motor aspects), and “to want” (motivational, volitional, and social readiness). Together, these components enable individuals to cope with training-related demands across variable situations (20–22). Applied to training, training competence is the set of prerequisites needed to meet the central demands of the training process with high training quality at both the process and the session level. Following recent conceptualizations of training quality, this includes designing and steering the overall training process (e.g., goal setting, gap analysis, and the deliberate application of training principles and methods) and optimizing individual sessions in line with their intended purpose, so that training is designed and executed to optimize adaptations and/or improve performance (7). To distinguish the session level from the process level of training, physical exercise—a single planned, structured, and purposive bout (i.e., one session)—is differentiated from physical training, defined as a planned, structured, and repetitive series of sessions conducted regularly over a defined period and aimed at improving or maintaining performance through longer-term adaptations (23–25).

Building on the training-process models of Hohmann et al. (26), Impellizzeri et al. (27), and Jeffries et al. (25), the present model captures the training process as a whole and derives the requirements individuals must meet to address its central demands with high training quality at both levels (7).

Physical training is thus conceptualized as a cyclical, feedback-driven process rather than a sequence of isolated sessions (26). It begins with the definition of training goals, proceeds via training prescription to the execution of a session, and returns through monitoring and evaluation to the refinement of goals and prescriptions. Consistent with Bucher Sandbakk et al. (7), this process can be divided into planning before the session, execution during the session, and evaluation after the session. This before-during-after logic distinguishes competence demands arising before (training goal-setting competence and training planning competence), during (training execution competence), and after (training monitoring and adjustment competence) training, yielding the four sub-competencies of the model.

Following Jeffries et al. (25), training prescription refers to the short- to long-term plans that define the nature and organization of training, whereas training load refers to the physical training actually done and experienced. External load is the work actually performed and is quantified independently of the individual's characteristics. Internal load is the individual's acute physiological, psychological, motor, and biomechanical response to the external load and to factors that amplify or attenuate the stimulus (11, 24, 28, 29); it is therefore a within-session construct, experienced during the session itself (27). Individual and contextual factors are not mere background variables but influences that can facilitate, constrain, or modify all major components of the process, including goals, prescription, load, training effects, and outcomes (25). Managing the external-internal load relationship is thus a core qualitative feature of training, because only an appropriately individualized external load is likely to elicit the intended internal response and, in turn, the desired training effects (27, 28). Training effects are the acute or chronic, positive or negative effects occurring after one or several sessions (25); they reflect the consequences of training exposure in performance-based, physiological, or subjective indicators (30). Goal-related outcomes are the distal outcomes of the process and indicate how far the intended goals have been achieved, for example sport performance in athletes or changes in physical capacity in recreational strength and endurance training. The feedback loop is closed through training monitoring and evaluation. Here, performance assessment (output) refers to isolated or periodic determinations of current status, whereas training documentation (input) denotes the ongoing recording of training-process information over time and thus supports monitoring (31). Evaluation integrates these data as a gap analysis between current and desired state and provides the basis for training adjustment (11, 26). Ultimately, the success of the process is judged against its outcomes, that is, the degree to which results match the predefined goals (8). On this basis, the model differentiates four competence domains: training goal-setting competence, training planning competence, training execution competence, and training monitoring and adjustment competence.

Competencies—and the sub-competencies derived from them—should be operationalized in a domain-specific manner, because what enables effective action in one area does not necessarily translate to another with different requirements (32). Following this logic, training competence should be differentiated across distinct training domains; in the present model, this is implemented by specifying competence separately for endurance and strength.

Training goal-setting competence comprises the ability to formulate appropriate short-, medium-, and long-term training goals. Individuals high in this competence define goals on the basis of their current performance status, relevant training-related knowledge, prior experience, and contextual constraints. This includes specifying desired outcomes in a realistic, measurable, and temporally structured way, thereby providing the basis for subsequent planning and evaluation.

Training planning competence comprises program design. Individuals with high training planning competence translate predefined goals into a coherent training prescription. This includes selecting and organizing appropriate training contents, methods, load parameters, and progression strategies over a defined period, while accounting for expected adherence, recovery demands, and relevant contextual constraints. It also includes anticipating control measures that allow subsequent evaluation of goal attainment and training quality. In line with the concept of control competence in the PAHCO model (32), it combines action-related knowledge (procedural knowledge about the planning, execution, and regulation of training) with effect-related knowledge (declarative knowledge about the expected effects of specific methods), enabling the purposeful application of training methods toward intended adaptations and outcomes. In endurance training, planning competence mainly requires prescribing and periodizing intensity, duration, and frequency, including the distribution of intensity across sessions (33). In strength training, it depends more strongly on manipulating load and volume variables, exercise selection and order, and rest intervals (34), implying partly distinct knowledge and skill requirements.

Training execution competence comprises body awareness and regulation. Individuals high in this competence carry out a session in accordance with its intended purpose and, where necessary, adapt it to current resources and situational demands. This includes perceiving relevant bodily and movement-related signals, such as fatigue, breathing, heart rate, perceived exertion, pain, or movement quality (35), and using them to regulate effort, pace, technique, and exercise execution in a goal-directed manner (36). It therefore entails flexible micro-adjustments when daily readiness, motivation, or contextual conditions deviate from what was anticipated, while minimizing technical errors, inappropriate overload, and other maladaptive responses (29). Its central function within the model is to regulate the performed external load so that it elicits the intended internal load. In endurance training, competent execution depends strongly on pacing, that is, task-specific, goal-directed regulation of effort that develops through practice and experience (37). In strength training, it relies more strongly on movement technique and on regulating proximity to failure, for example through repetitions-in-reserve judgments (38).

Training monitoring and adjustment competence comprises performance assessment, training documentation, and adjustment of the training process. Individuals high in this competence implement, interpret, and use control measures at the beginning of a training process and throughout its course. This includes knowing appropriate methods of performance assessment, applying them independently or seeking qualified support where necessary, and systematically documenting training contents, methods, and relevant load- and response-related parameters over time. Crucially, these data must be interpreted relative to session intentions, training effects, individual and contextual conditions, and goal-related outcomes in order to evaluate the gap between current and desired state and to derive justified adjustments to subsequent training (26). This competence thus extends beyond mere data collection to include evaluative judgment and corrective action. In both endurance and strength training, systematic documentation is used to track planned and completed sessions (30), but the emphasis of monitoring differs by modality. In endurance training, it focuses more strongly on intensity control and cardiorespiratory markers, such as pace, heart rate, and perceived exertion. In strength training, it centers more on workload and progression indicators, such as load lifted, repetitions completed, sets performed, and cumulative volume load (31).

Although analytically distinguishable, these four competence domains are recursively linked: goal setting defines the intended outcomes of the training process, planning translates these goals into a coherent prescription, execution generates the load and effects to be monitored, and monitoring and evaluation feed back into the ongoing adaptation of goals and prescriptions.

### 1.2 Goals of the study

The present study aimed to develop and provide initial validity evidence for a self-report measure of training competence in strength and endurance training, thereby addressing a conceptual and diagnostic gap in existing instruments. Existing instruments do not adequately capture training competence across the full training process as conceptualized here. In particular, related measures such as control competence within the PAHCO framework were primarily developed for health-enhancing physical activity and do not sufficiently differentiate key components of the training process from a training-science perspective, such as explicit goal setting, training prescription, the regulation of training execution and load, the monitoring of training effects, and the adjustment of training over time (15). The present instrument was therefore designed to operationalize four dimensions derived from the model: training goal setting competence, training planning competence, training execution competence, and training monitoring and adjustment competence. These dimensions were assumed to load on a higher-order factor reflecting overall training competence in endurance or strength training, while remaining applicable across both recreational and competitive contexts.

The study followed a phased development and validation approach in line with established recommendations for questionnaire construction and psychometric evaluation (39–41). First, the relevant constructs were specified and an expert-reviewed item pool was developed. Second, the scale was evaluated using a split-sample EFA-CFA cross-validation approach, complemented by analyses of reliability and discriminant and convergent validity.

## 2 Methods

### 2.1 Participants and data acquisition

A convenience sample of 1,454 young adults from nine secondary schools in north-west Germany were surveyed using an online questionnaire from September to December 2024. Prior to participation, written approval was obtained from the responsible school authorities. For participants who were under the age of legal consent, written parental consent was obtained in addition to participants' assent; participation was voluntary and students could decline to take part without any disadvantages. The study protocol was reviewed and approved by the Ethics Committee of the University of Vechta (approval date: November 2024), and all study procedures were conducted in accordance with the principles of the Declaration of Helsinki (42). Data collection was implemented by trained assessment teams who received standardized instruction as part of the research project, covering the conceptual background of the study as well as the applied procedures. The questionnaire was completed on site in the classrooms, so that the teams were available to answer questions and deal with any problems. Participants completed the questionnaire at their own pace, as no specific time limit was imposed. A total of 243 records were excluded because respondents discontinued the questionnaire before completion. Because the survey was cognitively demanding (e.g., extensive response formats with large item boxes) and required a comparatively long completion time (approximately 35–45 min), specific measures were implemented to safeguard response quality. Given the survey's length and cognitive demands, four evenly spaced attention checks were included as explicit instructed response items that required participants to select a predefined response option. This ex ante procedure helps detect inattentive responding, which can otherwise bias inter-item correlation and distort factor-analytic results (43). Participants were retained if they answered at least three of four attention checks correctly; 149 cases were excluded for failing this criterion. A further 23 participants were removed due to a self-reported condition preventing regular sport/exercise or insufficient German proficiency, yielding a final analytic sample of *n* = 1,039. The final analytic sample contained no missing values, because item nonresponse was prevented by the survey design: participants could proceed to the next page only after responding to all items displayed on the current page.

### 2.2 Development of the training competence questionnaire for endurance and strength (TCQ-E/S)

Guided by the training-process framework, the overarching construct of training competence and its dimensions were specified and refined through structured discussions in a focus group comprising three exercise scientists, two sport psychologists, and one expert in questionnaire design. This process comprised three structured review rounds, in which the item pool was iteratively evaluated for content relevance, clarity, and fit with the intended sub-competence, and items were revised, reformulated, or removed accordingly. For example, an initially double-barreled planning item (“I can plan my endurance training so that I include enough recovery and prevent injuries”) was considered to combine two distinct aspects that could be endorsed for different reasons, and to be neither specific enough nor sufficiently grounded in training-science terms; it was therefore reformulated into a single, clearly focused and exercise-science-based item (“I can plan my endurance training so that I include sufficient recovery between sessions to avoid excessive strain”). In parallel, the exercise science and sport psychology literature was screened for conceptually related constructs and existing instruments; findings from the literature review were integrated with the focus group input and iteratively refined through author discussions until consensus definitions were reached. This process resulted in a model with four dimensions—setting a training goal, training planning, training execution, and training monitoring/adjustment—each specified separately for endurance and strength, reflecting that these areas rely on partly distinct principles and knowledge bases. Building on these definitions, an initial item pool was drafted and then iteratively reviewed by the expert group with a primary focus on content validity and relevance, while also addressing clarity and wording. The resulting preliminary itempool comprised 122 items distributed across the eight dimensions: setting a training goal (endurance *n* = 7; strength *n* = 7), planning (endurance *n* = 24; strength *n* = 24), execution (endurance *n* = 12; strength *n* = 12), and monitoring/adjustment (endurance *n* = 18; strength *n* = 18). One item was adopted from the control competence scales of the PAHCO framework (16), assessing whether respondents know how to improve their endurance most effectively. Several additional items from this rather general and health-oriented scale were adapted by specifying them for endurance and strength training and by reformulating them in more training-science-specific terms. For the planning competence dimension, item development was additionally informed by key training principles, such as overload and progression (44), and items were formulated specifically for each domain. All further items were developed on the basis of the conceptual definitions and subcomponents of the respective subcompetence dimensions. Items were rated on a six-point Likert-type agreement scale ranging from strongly disagree to strongly agree. As training-related terminology (e.g., training method) cannot be assumed to be universally known, brief standardized explanations were provided immediately before the relevant item blocks. The item blocks were presented in a conceptually ordered sequence, whereas items were randomized within each block to reduce order effects.

### 2.3 Statistical analysis

All analyses were conducted using R Statistical Software version 4.4.2 and the packages lavaan version 0.6.21, psych version 2.5.6, semTools version 0.5.7 and performance version 0.15.0. Unless stated otherwise, statistical significance was set at *α* = .05. For cross-validation of the scale structure, the eligible dataset was randomly split into two subsamples (Table 1), with the first subsample used for exploratory factor analysis (EFA; *n* = 692) and the second subsample reserved for confirmatory factor analysis (CFA; *n* = 347).

**Table 1:** Participant characteristics.

Sample	Age (years)		BMI (kg/m^2^)		Sex		Sportsclub membership		Active in sports		Sportsindex if active (minutes)	
	*M*	SD	*M*	SD	Male	Female	Yes	No	Yes	No	*M*	SD
EFA sample (*n* = 692)	18.39	2.94	22.64	3.81	38.29%	61.71%	55.78%	44.22%	71.24%	28.76%	305.11	208.07
CFA sample (*n* = 347)	18.28	2.63	22.16	3.24	38.61%	61.39%	57.64%	42.36%	70.60%	29.40%	289.01	194.46
Overall (*n* = 1.039)	18.35	2.84	22.48	3.63	38.40%	61.60%	56.40%	43.60%	71.02%	28.98%	299.77	203.66
Retest sample (*n* = 99)	16.52	0.60	22.61	3.77	48.48%	51.52%	56.56%	43.43%	59.59%	40.41%	283.05	229.78

*M*, mean; SD, standard deviation.

Both sample sizes exceed commonly cited benchmarks for applied factor-analytic validation (*n* ≈ 300), supporting stable factor recovery and a robust cross-validation of the measurement model (40). Random splitting yielded comparable EFA and CFA subsamples, with no significant differences in age, sex, sports club membership, current sport participation, or sport-index scores (*p* > .30 for all comparisons). BMI differed only nominally and did not remain significant after correction for multiple comparisons, supporting the use of the two subsamples for cross-validation. Temporal stability was evaluated using a two-week test-retest design in an independent subsample (*n* = 99), based on the shortened CFA-derived version of the scale (41). Compared with the EFA and CFA subsamples, the retest sample was younger, whereas group differences in sex, BMI, sports club membership, and sport-index scores were not significant; nominal differences in current sport participation did not remain significant after correction.

#### 2.3.1 Exploratory factor analysis

Within the EFA subsample, the initial item pool was refined iteratively using predefined criteria, with conceptual interpretability as the primary decision rule. Factorability was examined using the Kaiser–Meyer–Olkin (KMO) measure of sampling adequacy and Bartlett's test of sphericity, and factors were estimated with maximum-likelihood extraction (ML) and oblimin rotation given the expectation of correlated factors and potential higher-order structure (40). To support the use of ML, univariate skewness and kurtosis were checked against common cutoffs (skewness < 2.0; kurtosis < 7.0) (45). Based on theory, an eight-factor solution was targeted, and factor retention was guided by Empirical Kaiser's Criterion, the Minimum Average Partial test (MAP), and Parallel Analysis, with discrepancies resolved via parsimony and interpretability (40, 46). Item retention balanced content coverage with key psychometric criteria: salient primary loadings (≥.50 preferred; ≥.30 minimum), limited cross-loadings (items were retained/assigned only when the primary loading exceeded the next-largest loading by >.10), adequate communalities (>.50 desirable; removal considered when <.40), non-extreme item difficulty (avoiding near-universal or very low endorsement), and corrected item–total correlations (≥.30 minimum; ≥.50 desirable). Items failing these criteria were removed stepwise and the EFA was re-estimated after each deletion until a simple structure was achieved with sufficiently represented factors (≥4 indicators) and adequate explained variance (≥60% considered desirable) (47).

#### 2.3.2 Confirmatory factor analysis

Following the exploratory phase, CFA was used to test the EFA-derived structure in a hypothesis-driven manner, with the factor number and item–factor assignments specified *a priori*. Models were estimated using robust maximum likelihood (MLR) with the Satorra–Bentler scaled *χ*^2^ and robust standard errors (40). Model fit was evaluated using complementary indices (RMSEA with CI, SRMR, CFI, TLI), while SB–*χ*^2^ was reported but not treated as the sole decision criterion due to its sensitivity to sample size and model complexity (41). The normalized chi-square (SB–*χ*^2^/df) was additionally considered, with values <2 interpreted as good and values <5 as acceptable (41). Commonly used benchmarks were applied as heuristic guidelines rather than rigid rules: RMSEA/SRMR ≤ .05 and CFI/TLI ≥ .95 were interpreted as indicative of good model fit, whereas RMSEA/SRMR ≤ .08 and CFI/TLI ≥ .90 were considered acceptable (41).

A hierarchical second-order CFA was then estimated within the same measurement framework to examine whether the first-order solution could be represented more parsimoniously by two correlated higher-order factors. All first-order factors were modeled simultaneously, with the endurance-related factors loading on a second-order endurance training competence factor and the strength-related factors loading on a second-order strength training competence factor. The model was estimated using the same MLR approach and compared with the first-order model using changes in global fit (ΔCFI ≤ .010, ΔRMSEA ≤ .015, ΔSRMR ≤ .030) as evidence of no meaningful loss of fit (48).

#### 2.3.3 Validity and reliability analysis

Convergent and discriminant validity were examined in the CFA model using the Fornell–Larcker approach by computing the average variance extracted (AVE) for each factor (convergent validity: AVE ≥ .50; discriminant validity: AVE greater than the squared correlation with any other factor) (49). Discriminant validity was additionally evaluated following Rönkkö and Cho (50) by scaling latent variables via fixed factor variances (=1) and using the upper bound (UL) of the 95% CI of each latent factor correlation as the decision-relevant criterion (UL < .80: no evidence of a concern;. 80 ≤ UL < .90: marginal concern; .90 ≤ UL < 1.00: moderate concern; ≥1.00: severe concern). Results in the marginal range indicate that interpreting the scales as representing distinct constructs is generally still warranted. As a further check, Wald tests assessed whether factor correlations differed from 1.00. Reliability was evaluated at factor and indicator level. In the EFA subsample, Cronbach's alpha was reported as a conventional internal consistency estimate, whereas in the CFA subsample construct-level reliability was quantified using composite reliability (≥.70 acceptable) (47). Across both subsamples, indicator reliability was assessed via squared multiple correlations (SMC) (≥.50 desirable) and average inter-item correlations (≥.30 indicative of good reliability) (47). Test–retest reliability was examined using the intraclass correlation coefficient (ICC) (two-way mixed-effects, single measurement, absolute agreement) and interpreted using commonly applied benchmarks (poor <.50, moderate .50–.75, good .75–.90, excellent >.90) (51).

#### 2.3.4 Measurement invariance

Measurement invariance across sex, current sport participation (yes/no), and sports club membership was tested in the combined sample using stepwise multi-group CFA: configural (same factor structure across groups), metric (additionally equal unstandardized factor loadings), and scalar (additionally equal item intercepts). Invariance across these three variables is relevant because the groups likely differ in training-related knowledge and experience and might therefore interpret the items differently. This applies, for example, to current sport participants and non-participants, or to club members with access to organized, coach-supported training vs. those training in informal, self-organized settings. Scalar invariance was interpreted as strong factorial invariance, supporting comparisons of observed scale means. Invariance was assumed when changes in fit remained within recommended thresholds [ΔCFI ≤ .010, ΔRMSEA ≤ .015, ΔSRMR ≤ .030; (48)].

## 3 Results

### 3.1 Exploratory factor analysis

Normality screening indicated no severe univariate non-normality (all items within the predefined cutoffs of skewness <2.0 and kurtosis <7.0), supporting the ML-based EFA. The correlation matrix was well suited for factor analysis [KMO = 0.983; Bartlett's test *χ*^2^(7,381) = 89,159.59, *p* < .001]. Factor-retention criteria diverged: Empirical Kaiser's Criterion suggested a 12-factor solution, Parallel Analysis indicated nine factors, and the MAP test supported eight factors. Therefore, consistent with the *a priori* theoretical expectation and recommendations to prioritize conceptual interpretability and parsimony when retention criteria suggest different factor solutions, an eight-factor solution was initially extracted for item screening and reduction (40). Stepwise removal of items not meeting predefined criteria reduced the pool from 122 to 62 items. In total, 60 items were discarded due to clear shortcomings, an insufficient number of interpretable indicators per extracted factor, salient loadings below .30, or problematic cross-loading patterns (i.e., loadings of similar magnitude with differences <.10 that were not conceptually justifiable). Although an eight-factor solution was initially targeted, inspection of the loading matrix indicated that two of the extracted factors were not well-defined (one factor showed only a single salient loading >.30 and no coherent loading pattern; another displayed a few high loadings but lacked an interpretable structure). This pattern was attributable to the fact that the theoretically proposed goal-setting dimensions did not emerge as separate factors; instead, the goal-setting items loaded primarily on the corresponding training-planning factors (endurance and strength), suggesting that goal setting was empirically embedded within training planning. Therefore, subsequent analyses proceeded with a six-factor extraction to improve parsimony and conceptual clarity. In the subsequent six-factor EFA, the proportion of explained variance remained at 65%, matching the earlier eight-factor extraction. The six-factor solution showed the intended simple structure with only a limited number of cross-loadings (see Supplementary Tables 1–6). Only one item showed a cross-loading that exceeded .10 and was subsequently removed and the model was re-estimated. In the final solution, standardized loadings ranged from .37 to .89, cross-loadings were minimal (no cross-loading >.30) and 65% of total variance were explained. The only notable overlap was for TES1 (loading .37 on Execution [Strength] and .29 on Monitoring/Adjustment [Strength]; Δ = .08), which was retained to preserve content coverage. Communalities (.41–.81), item difficulty (.54–.77), and corrected item–total correlations (>.63) indicated good factor representation, adequate response variability, and strong item–scale coherence.

### 3.2 Confirmatory factor analysis

The CFA tested the six-factor first-order measurement model (training planning, execution, and monitoring/adjustment; each specified for endurance and strength). Standardized factor loadings were uniformly positive and substantial, ranging from .63 to .89 across all indicators (average loading ≈.77). Model fit of the six-factor first-order solution was acceptable: SB-*χ*^2^(1,754) = 3,218.41, *p* < .001, SB-*χ*^2^/df = 1.83, CFI = .912, TLI = .908, RMSEA = .054 (90% CI .051–.057), and SRMR = .051 (see Table 2). A more parsimonious second-order model with two higher-order factors was subsequently tested and also showed acceptable fit: SB-*χ*^2^(1,762) = 3,377.94, *p* < .001, SB-*χ*^2^/df = 1.92, CFI = .902, TLI = .899, RMSEA = .057 (90% CI .054–.060), and SRMR = .059. Relative to the first-order model, fit decreased only marginally (ΔCFI = .010, ΔRMSEA = .003, ΔSRMR = .008), indicating no practically meaningful loss of fit when moving to the hierarchical representation.

**Table 2:** Model fit indices of tested confirmatory factor analysis models.

Factor solution	SB-*χ*^2^	df	*χ*^2^/df	*p*	CFI	TLI	RMSEA	LO90	HI90	SRMR	ΔCFI	ΔRMSEA	ΔSRMR
Six first-order-factors	3,218.412	1,754	1.834	.000	0.912	0.908	0.054	0.051	0.057	0.051			
Two second-order-factors	3,377.936	1,762	1.917	.000	0.902	0.899	0.057	0.054	0.060	0.059	0.100	0.003	0.008

Robust (MLR-scaled) fit indices are reported. SB-*χ*^2^, Satorra-Bentler scaled chi-square; df, degrees of freedom; CFI, comparative fit index; TLI, Tucker Lewis Index; RMSEA, root mean square error of approximation; LO90, lower boundary of 90% confidence interval; HI90, upper boundary of 90% confidence interval; SRMR, standardized root mean square residual; Δ, difference of a parameter to first-order solution.

### 3.3 Validity and reliability analysis

Convergent validity was supported, with AVE values above .50 for all six factors (range .58–.72). Using Fornell–Larcker, discriminant validity was supported for five factors; the only exception was the closely related pair Execution (Strength) and Training Planning (Strength), where shared variance (.65) exceeded AVE (.58), indicating overlap for this specific comparison (see Supplementary Table 8). Using the Rönkkö and Cho (50) confidence-interval approach, 11 of 15 factor pairs showed no evidence of a discriminant validity problem (UL < .80), and the remaining four were in the marginal range (.80 ≤ UL < .90), with no moderate or severe cases. Wald tests further rejected perfect correlations between factors. Reliability indices were consistently strong: Cronbach's *α* (EFA) and composite reliability (CFA) were uniformly high (both .90–.98), average inter-item correlations indicated substantial within-scale homogeneity (≈.56–.74), and indicator reliability (SMC) ranged from .39 to .80. Test–retest ICCs indicated moderate-to-good temporal stability (.74–.85), with the lowest value observed for Planning–Endurance (ICC = .74) (see Supplementary Tables 7, 8).

### 3.4 Measurement invariance

Multi-group CFA supported scalar invariance of the six-factor model across sex, current sport activity, and sports club membership (see Supplementary Table 9). Configural models indicated the same factor structure across groups, and adding equality constraints on factor loadings (metric) and item intercepts (scalar) produced only negligible changes in model fit (all ΔCFI, ΔRMSEA, and ΔSRMR within the predefined thresholds), permitting comparisons of observed scale means between groups.

## 4 Discussion

This study developed the TCQ-E/S and provided initial evidence for its factorial, convergent, and discriminant validity, as well as reliability at the item and construct level in young adults. Overall, the findings supported the TCQ-E/S as a multidimensional self-report instrument capturing key demands of self-managed training across planning, execution, and monitoring/adjustment, specified separately for endurance and strength.

Regarding internal structure, iterative item reduction (from an initial pool of 122 items to 62 items, followed by the removal of one additional cross-loading item) produced a clear six-factor simple-structure solution explaining 65% of the variance, with predominantly salient loadings and only few, small cross-loadings. This yielded a six-factor structure that mapped directly onto the proposed framework, reflecting the intended separation of endurance and strength across the three core training-process components. Notably, the initially hypothesized goal-setting facets did not emerge as distinct factors; instead, goal-setting items loaded on the respective planning factors. This suggests that, in the present operationalization, goal definition is empirically embedded within training planning—a plausible pattern given that goal setting, gap analysis, and program design are closely intertwined in self-regulated training (26). A likely reason is that both draw on largely the same knowledge base, as formulating a realistic training goal already requires the knowledge needed to plan the corresponding training. The CFA in an independent subsample supported the EFA-derived structure, with consistently substantial standardized item loadings and overall acceptable model fit for the comparatively large item set. As theoretically expected, the six first-order factors were also well represented by a hierarchical structure with two second-order factors reflecting endurance and strength training competence. This higher-order pattern is conceptually plausible because endurance and strength training draw on partly distinct training principles and knowledge bases for key demands of the training process while retaining the same overarching process architecture. Convergent validity was supported, as AVE values exceeded .50 for all constructs. Evidence for discriminant validity was largely favorable, with one notable indication of overlap between two closely related facets. Under the Fornell-Larcker criterion, Execution (Strength) shared substantial variance with Training Planning (Strength), indicating a close relationship between these facets. Because the AVE-based Fornell-Larcker comparison is a relatively stringent, dichotomous heuristic that can flag borderline overlap, greater interpretive weight was placed on the confidence-interval-based guideline proposed by Rönkkö and Cho (50), which treats discriminant validity as a matter of degree. Accordingly, the planning–execution (strength) association was classified as at most a marginal concern, which their interpretive guidance considers generally compatible with treating the scales as empirically distinct.

Across subscales, internal consistency was high, as reflected by Cronbach's alpha in the EFA subsample and composite reliability in the CFA subsample, with average inter-item correlations likewise supporting strong within-scale homogeneity. Importantly, multi-group CFA supported scalar measurement invariance across sex, sports club membership, and current sport participation, indicating that the items functioned equivalently across these subgroups (i.e., they reflected the same structure and had comparable item–factor relations and intercepts). This suggests that the items were understood in the same way and were similarly informative for the respective sub-competencies, even though some dimensions include knowledge-related components that could differ by training experience; thus, between-group mean differences can be interpreted as substantive rather than measurement-driven. In addition, test–retest reliability over a two-week interval was moderate to good, indicating adequate temporal stability of scores and thereby supporting the use of the TCQ-E/S in longitudinal contexts.

Several limitations should be noted. The sample consisted entirely of young adults recruited from secondary schools in a single region of Germany. Generalizability to other groups, including experienced adult exercisers, older adults, and competitive or elite athletes, therefore remains to be established, and the instrument's measurement invariance across broader age ranges and performance levels was not examined. Beyond sample composition, a further consideration concerns the training demands the instrument represents: with increasing training experience and performance level, the training process often relies on more differentiated methods of training planning, regulation, and monitoring, such as systematic periodization, detailed load management, and physiological or performance-based control, which the present items, developed for self-directed training in young recreational exercisers, may not fully capture. Consequently, the TCQ-E/S may underrepresent the competence demands relevant in more advanced or specialized training contexts, and its factorial structure should be re-examined before it is applied in these populations. The same caveat applies to other countries and cultures: because the instrument was developed and validated in German, and because national systems of physical education, sport participation, and access to structured training may vary, item interpretation and measurement equivalence should not be taken for granted across languages and cultural settings, so that translation, cross-cultural adaptation, and further validation would be advisable before broader application. The high internal consistency observed for the subscales, together with the length of the instrument, may partly reflect item redundancy. Developing and validating shorter versions could therefore address such potential redundancy while increasing the feasibility and applicability of the instrument for applied assessment and large-scale research.

Furthermore, as a self-report measure, the TCQ-E/S primarily captures perceived training competence; criterion-related validity therefore still needs to be established by examining its associations with external, preferably more objective criteria (e.g., performance on domain-specific training knowledge tests, relevant fitness outcomes, or expert ratings of self-developed training plans). For example, participants could design a training plan whose quality is rated by independent coaches or experts, following the procedure recently applied by Havers et al. (52) to AI-generated hypertrophy and strength plans, with these ratings then related to self-assessed training competence. The inclusion of fitness outcomes among these criteria is likewise supported by PAHCO research, in which control competence for physical training was positively associated with objectively assessed health-related fitness (endurance and strength), independent of habitual sport activity, in line with the present definition of competence as the application of action- and effect-related knowledge to training demands (53). Beyond examining such criterion-related links, future studies should also aim to assess training competence objectively, because self-evaluations are often imprecise and can involve systematic over- or underestimation (54). Accordingly, the PAHCO literature has explicitly called for performance-based competence assessments to complement self-report indicators of control competence (16). As an example for endurance contexts, Thiel et al. (55) propose pacing-based tasks as a promising way, because pacing can be evaluated against observable criteria of appropriate effort regulation. In addition, prospective longitudinal designs and, ideally, randomized controlled trials are needed to test whether TCQ-E/S scores predict subsequent training-process quality and, in turn, meaningful training outcomes such as fitness or performance improvements, adherence, and injury risk.

The present study also has several strengths. First, the TCQ-E/S is grounded in a theoretically derived, multidimensional model of the training process that differentiates competencies separately for endurance and strength, a distinction that was also empirically supported by the factor-analytic results. Second, the instrument was developed and evaluated in a large sample, enabling a split-sample design with independent exploratory and confirmatory factor analyses and supporting validity and reliability across multiple lines of evidence, including measurement invariance and test-retest stability.

These properties translate into concrete practical applications. Because the TCQ-E/S yields a differentiated profile across the sub-competencies and across the endurance and strength domains, rather than a single global score, it can be used to identify where an individual's support needs lie. For example, a person scoring high on planning competence but low on execution competence would be expected to design appropriate training yet struggle to implement sessions as intended; support should then target session execution, such as perceiving and regulating effort, pace, or technique, rather than program design. Such profiles can inform practice in settings in which self-directed training is prevalent, such as commercial fitness and health facilities, sports clubs, university and school sport, athlete education, and exercise-based rehabilitation, including digitally delivered formats. In these settings, the TCQ-E/S could serve as a needs-assessment tool to identify individual competence gaps and to tailor educational or supervisory support, or as an outcome measure to evaluate competence-oriented interventions. Integrated into a training programme, it could be administered at intake to match the type and intensity of support to the individual profile, for instance by providing closer instruction where competence is low and greater autonomy where it is high, and repeatedly over the course of a programme to track competence development and adjust educational input accordingly.

Initial research is likewise needed to determine how and in what settings training competence can be promoted. First evidence in school settings has been provided by Thienes et al. (56), who frame training competence as a health resource because it is intended to enable individuals to organize their own training autonomously and in a self-regulated manner, thereby supporting improvements in motor performance and physical fitness. This aligns with developmental models proposing reciprocal dynamics between motor competence/fitness and physical activity, suggesting that competence-supported gains in fitness may, in turn, facilitate sustained engagement over time (57).

## 5 Conclusion

In sum, the findings support the TCQ-E/S as a theoretically grounded, multidimensional self-report instrument for assessing individual training competence in endurance and strength across planning, execution, and monitoring/adjustment. The cross-validated factor structure, subgroup invariance, and temporal stability indicate that the instrument is suitable for between-group comparisons and has promising potential for use in longitudinal research. Beyond research applications, the TCQ-E/S may be useful in gyms and fitness settings, club and competitive sport (including decentralized training contexts), and educational settings (e.g., physical education) to identify individual support needs, inform targeted instruction or counseling, stimulate reflection on one's own training process, and evaluate interventions aimed at fostering training competence.

## Data Availability

The datasets analyzed in this study are available from the corresponding author upon reasonable request.
