# Supplementary material for: Training competence questionnaire for endurance and strength training: development and validation
Source: Front Sports Act Living. 2026 Aug 7;8:1909973. doi: 10.3389/fspor.2026.1909973 (PMC13505200; doi:10.3389/fspor.2026.1909973)
Supplement: Supplementary file 1 [file Table1.docx]

Supplementary Material

|  | **Item** | **M** | **SD** | **r_it_** | **SMC** | **Factor** | | | | | |
| --- | --- | --- | --- | --- | --- | --- | --- | --- | --- | --- | --- |
|  |  |  |  |  |  | **1** | **2** | **3** | **4** | **5** | **6** |
| TPETG1 | I am able to set appropriate and realistic goals for my endurance training. | 4.08 | 1.16 | .65 | .42 |  | .58 |  |  |  |  |
| TPETG5 | I am able to prioritize my training goals to ensure that I focus on the most important aspects of my endurance training. | 4.25 | 1.00 | .71 | .51 |  | .67 |  |  |  | .15 |
| TPETG6 | I am able to adjust my training goals after endurance training if I realize that they are not realistic or appropriate. | 4.42 | 1.06 | .63 | .41 |  | .66 |  |  |  |  |
| TPETG8 | I can set appropriate and realistic short- and long-term goals for my endurance training. | 4.05 | 1.06 | .78 | .61 |  | .78 |  |  |  | .11 |
| TPE7 | I can plan my endurance training so that I avoid excess or insufficient load. | 4.11 | 1.06 | .72 | .51 |  | .66 |  |  |  |  |
| TPE8 | I make sure my workout routine follows a logical sequence to maximize its effectiveness. | 3.89 | 1.19 | .71 | .53 |  | .63 | .19 |  |  |  |
| TPE9 | I can plan my endurance training so that I include sufficient recovery between sessions to avoid excessive strain. | 4.31 | 1.10 | .65 | .43 |  | .66 |  |  | .13 |  |
| TPE10 | I can plan my endurance training so that it becomes increasingly challenging as I make progress. | 4.10 | 1.07 | .82 | .67 |  | .76 |  |  |  |  |
| TPE11 | I am familiar with various types of endurance training exercises and can choose them specifically to meet my training goals. | 3.85 | 1.19 | .81 | .67 |  | .75 |  |  |  |  |
| TPE14 | I can plan my endurance training to include regular sessions so that I can keep improving. | 3.99 | 1.10 | .84 | .72 |  | .79 |  |  |  |  |
| TPE15 | I can choose endurance training methods in such a way that I train exactly what I intend to. | 4.02 | 1.11 | .80 | .66 | .13 | .73 |  |  |  |  |
| TPE16 | I can vary the content and methods of my endurance training over extended periods of time, for example, to maximize my progress or maintain my motivation. | 3.89 | 1.15 | .82 | .68 |  | .76 |  |  |  |  |
| TPE18 | I know how to use physical training to improve my endurance in the best possible way. | 4.02 | 1.16 | .74 | .56 |  | .63 |  |  |  |  |
| TPE20 | I can create an endurance training plan that takes both short-term and long-term training goals into account. | 3.76 | 1.18 | .80 | .67 |  | .76 | .13 |  |  |  |
| TPE21 | I can tailor my endurance training activities (e.g., content and methods) to my training goals. | 3.91 | 1.11 | .84 | .72 |  | .74 | .11 |  |  |  |
| TPE24 | I am able to plan my endurance training in a way that takes my individual readiness and performance capabilities into account. | 4.23 | 1.08 | .76 | .58 |  | .66 |  | .11 |  |  |

Table 1. Descriptive statistics and factor loadings for the factor training planning endurance.

Notes: TPE = Training planning endurance; TPEPG = Training planning endurance training goal; M = mean; SD = standard deviation; r_it_ = item–total correlation; SMC = squared multiple correlation. Factor loadings based on the exploratory factor analysis; only loadings ≥ .10 are displayed.

Table 2. Descriptive statistics and factor loadings for the factor training planning strength.

|  | **Item** | **M** | **SD** | **r_it_** | **SMC** | **Factor** | | | | | |
| --- | --- | --- | --- | --- | --- | --- | --- | --- | --- | --- | --- |
|  |  |  |  |  |  | **1** | **2** | **3** | **4** | **5** | **6** |
| TPSTG1 | I am able to set appropriate and realistic goals for my strength training. | 4.10 | 1.27 | .84 | .70 | .79 |  |  |  |  |  |
| TPSTG5 | I am able to prioritize my training goals to ensure that I focus on the most important aspects of my strength training. | 4.21 | 1.17 | .84 | .72 | .66 | .20 |  |  | .10 |  |
| TPSTG6 | I am able to adjust my training goals after strength training if I realize that they are not realistic or appropriate. | 4.28 | 1.21 | .78 | .62 | .71 |  |  |  | .17 |  |
| TPSTG8 | I can set appropriate and realistic short- and long-term goals for my strength training. | 4.03 | 1.19 | .87 | .76 | .73 |  |  |  |  |  |
| TPS3 | I am able to plan my strength training such that the sequence of exercises and training components is appropriately structured. | 4.11 | 1.27 | .85 | .73 | .83 |  |  |  |  |  |
| TPS6 | I am able to regularly adjust my strength training to ensure that it remains challenging and continues to promote progress. | 4.20 | 1.22 | .86 | .76 | .83 |  |  |  |  |  |
| TPS7 | I can plan my strength training so that I avoid excess or insufficient load. | 4.20 | 1.22 | .79 | .64 | .75 |  |  | .12 |  | .10 |
| TPS12 | I am able to select training exercises for my strength training in a way that targets exactly what I intend to train. | 4.14 | 1.31 | .88 | .78 | .89 |  |  |  |  |  |
| TPS13 | I am able to plan my strength training in a way that allows for sufficient recovery between training sessions to avoid excessive load. | 4.26 | 1.26 | .76 | .59 | .75 |  |  |  | .10 |  |
| TPS14 | I can plan my strength training to include regular sessions so that I can keep improving. | 4.05 | 1.26 | .89 | .79 | .82 |  |  |  |  |  |
| TPS15 | I can choose strength training methods in such a way that I train exactly what I intend to. | 4.11 | 1.29 | .88 | .79 | .89 |  |  |  |  |  |
| TPS16 | I can vary the content and methods of my strength training over extended periods of time, for example, to maximize my progress or maintain my motivation. | 3.98 | 1.27 | .86 | .75 | .80 |  |  |  |  |  |
| TPS18 | I know how to use physical training to improve my strength in the best possible way. | 4.10 | 1.27 | .82 | .69 | .63 |  | .17 |  |  |  |
| TPS20 | I can create a strength training plan that takes both short-term and long-term training goals into account. | 3.85 | 1.27 | .84 | .73 | .69 | .11 | .13 |  |  |  |
| TPS21 | I can tailor my strength training activities (e.g., content and methods) to my training goals. | 4.00 | 1.26 | .89 | .80 | .81 |  | .13 |  |  |  |
| TPS22 | I am able to plan my strength training in a way that matches my current fitness level and training experience. | 4.18 | 1.27 | .87 | .76 | .87 |  |  |  |  |  |

Notes: TPS = Training planning strength; TPSPG = Training planning strength training goal; M = mean; SD = standard deviation; rit = item–total correlation; SMC = squared multiple correlation. Factor loadings based on the exploratory factor analysis; only loadings ≥ .10 are displayed.

Table 3. Descriptive statistics and factor loadings for the factor training execution endurance.

|  | **Item** | **M** | **SD** | **r_it_** | **SMC** | **Factor** | | | | | |
| --- | --- | --- | --- | --- | --- | --- | --- | --- | --- | --- | --- |
|  |  |  |  |  |  | **1** | **2** | **3** | **4** | **5** | **6** |
| TEE1 | I am able to estimate when my cardiovascular system will become fatigued during endurance training. | 4.11 | 1.17 | .74 | .57 |  |  |  | .69 | .11 |  |
| TEE2 | I am able to use my heart rate or breathing to adjust my training intensity (e.g., to go faster or slower). | 4.04 | 1.22 | .79 | .68 |  |  |  | .77 |  |  |
| TEE3 | I am able to assess my load during endurance training based on my heart rate and breathing. | 4.04 | 1.15 | .81 | .73 |  |  |  | .82 |  |  |
| TEE4 | I am able to adjust my endurance training (e.g., by going faster or slower) when I perceive that my internal load is too high or too low. | 4.36 | 1.11 | .78 | .60 |  | .10 |  | .57 | .17 | .16 |
| TEE5 | I am able to recognize signs of fatigue during endurance training. | 4.48 | 1.07 | .75 | .56 |  |  |  | .59 | .27 |  |
| TEE6 | When I perceive a decline in movement quality during endurance training, I am able to adjust my training load accordingly (e.g., by modifying pace). | 4.33 | 1.10 | .79 | .60 |  | .16 |  | .54 | .21 |  |
| TEE12 | I am able to perceive declines in movement quality (e.g., running or swimming technique) during endurance training. | 4.28 | 1.09 | .63 | .44 |  | .20 |  | .38 | .24 | .16 |

Notes: TEE = Training execution endurance; M = mean; SD = standard deviation; rit = item–total correlation; SMC = squared multiple correlation. Factor loadings are based on the exploratory factor analysis; only loadings ≥ .10 are displayed.

Table 4. Descriptive statistics and factor loadings for the factor training execution strength.

|  | **Item** | **M** | **SD** | **r_it_** | **SMC** | **Factor** | | | | | |
| --- | --- | --- | --- | --- | --- | --- | --- | --- | --- | --- | --- |
|  |  |  |  |  |  | **1** | **2** | **3** | **4** | **5** | **6** |
| TES1 | I am able to estimate when I will reach muscle failure during an exercise. | 4.29 | 1.28 | .76 | .60 | .25 |  | .29 | .19 | .37 |  |
| TES2 | If my current readiness does not allow me to achieve the planned training load, I adjust it accordingly. | 4.28 | 1.24 | .71 | .53 | .15 |  |  | .14 | .51 |  |
| TES3 | I am able to perceive signs of muscular fatigue. | 4.61 | 1.10 | .80 | .61 |  |  | .16 | .17 | .60 |  |
| TES6 | I am able to recognize when my muscular strength decreases. | 4.67 | 1.11 | .76 | .57 |  |  | .14 | .15 | .59 |  |
| TES7 | I am able to adjust my training load based on my level of muscular fatigue (e.g., by modifying weight or repetitions). | 4.38 | 1.20 | .86 | .76 | .11 |  | .20 |  | .64 |  |
| TES8 | I am able to recognize the point at which no further repetition can be performed with proper technique. | 4.29 | 1.24 | .76 | .59 | .23 |  | .11 | ..10 | .46 | .13 |
| TES10 | If the prescribed training load is too low or too high for me, I am able to adjust it accordingly (e.g., by modifying the weight). | 4.46 | 1.16 | .86 | .75 | .28 |  |  |  | .60 |  |
| TES11 | I am able to recognize when the prescribed training weight is too low or too high for me. | 4.45 | 1.17 | .82 | .68 | .28 |  | .10 | .11 | .51 |  |

Notes: TES = Training execution strength; M = mean; SD = standard deviation; rit = item–total correlation; SMC = squared multiple correlation. Factor loadings are based on the exploratory factor analysis; only loadings ≥ .10 are displayed.

Table 5. Descriptive statistics and factor loadings for the factor training monitoring and adjustment endurance.

|  | **Item** | **M** | **SD** | **r_it_** | **SMC** | **Factor** | | | | | |
| --- | --- | --- | --- | --- | --- | --- | --- | --- | --- | --- | --- |
|  |  |  |  |  |  | **1** | **2** | **3** | **4** | **5** | **6** |
| TME2 | I am familiar with different testing procedures for assessing my endurance performance. | 3.29 | 1.41 | .76 | .58 |  |  | .15 | .11 |  | .61 |
| TME6 | I know which indicators are relevant for assessing and evaluating my endurance performance. | 3.80 | 1.23 | .77 | .58 | .14 |  |  | .13 |  | .60 |
| TME11 | I am able to adjust my endurance training when my training evaluations show that I am not achieving my goals. | 3.87 | 1.23 | .73 | .60 | .10 | .25 |  | .13 |  | .50 |
| TME12 | I am able to independently evaluate my current endurance performance using appropriate testing procedures. | 3.44 | 1.38 | .82 | .70 |  |  | .19 | .11 |  | .67 |
| TME16 | I am able to evaluate, on the basis of data from performance assessments and training documentation, whether my current endurance training is effective. | 3.63 | 1.30 | .82 | .69 |  |  |  |  |  | .71 |
| TME17 | If I intend to document my endurance training, I know how and with which tools or methods to do so. | 3.67 | 1.43 | .79 | .63 |  |  | .13 |  |  | .69 |
| TME18 | I am able to document my endurance training (e.g., in writing or using an app). | 3.96 | 1.47 | .71 | .56 |  |  |  |  | .18 | .69 |

Notes: TEE = Training monitoring endurance; M = mean; SD = standard deviation; rit = item–total correlation; SMC = squared multiple correlation. Factor loadings are based on the exploratory factor analysis; only loadings ≥ .10 are displayed.

Table 6. Descriptive statistics and factor loadings for the factor training monitoring and adjustment strength.

|  | **Item** | **M** | **SD** | **r_it_** | **SMC** | **Factor** | | | | | |
| --- | --- | --- | --- | --- | --- | --- | --- | --- | --- | --- | --- |
|  |  |  |  |  |  | **1** | **2** | **3** | **4** | **5** | **6** |
| TMS2 | I am able to independently evaluate my current strength performance using appropriate testing procedures. | 3.57 | 1.44 | .86 | .75 |  |  | .73 |  |  | .10 |
| TMS5 | I am able to adjust my strength training based on my training evaluations in order to achieve better results. | 3.87 | 1.39 | .83 | .70 | .16 |  | .57 |  | .15 | .11 |
| TMS6 | I know which indicators are relevant for assessing and evaluating my strength performance. | 3.80 | 1.33 | .85 | .73 | .10 |  | .65 |  | .11 |  |
| TMS12 | I know how to effectively document my strength training activities. | 3.61 | 1.44 | .89 | .79 |  |  | .76 |  |  | .10 |
| TMS13 | I am familiar with different testing procedures for assessing my strength performance. | 3.44 | 1.44 | .86 | .78 |  |  | .75 |  |  | .12 |
| TMS14 | I am able to evaluate, on the basis of data from performance assessments and training documentation, whether my current strength training is effective. | 3.68 | 1.41 | .90 | .81 |  |  | .70 |  | .13 | .10 |
| TMS15 | I am able to document my strength training (e.g., in writing or using an app). | 3.88 | 1.44 | .83 | .70 |  |  | .61 |  | .19 | .16 |

Notes: TMS = Training monitoring strength; M = mean; SD = standard deviation; rit = item–total correlation; SMC = squared multiple correlation. Factor loadings are based on the exploratory factor analysis; only loadings ≥ .10 are displayed.

Table 7. Internal consistency and average inter-item correlations for the subscales of training competence in endurance and strength.

| **Factor** | **Cronbachs α** | **Average inter-item correlation** |
| --- | --- | --- |
| Training planning endurance | 0.95 | 0.56 |
| Training planning strength | 0.98 | 0.71 |
| Training execution endurance | 0.90 | 0.57 |
| Training execution strength | 0.93 | 0.62 |
| Training monitoring and adjustment endurance | 0.91 | 0.59 |
| Training monitoring and adjustment strength | 0.95 | 0.74 |

Table 8. Factor loadings, reliability indices, and validity indicators for the training competence subscales in endurance and strength.

| **Skala** | **Factor loadings** | **Indiciator reliability** | **Composite reliability** | **Average inter-item correlation** | **Average variance extracted** | **Highest squared correlation** | **Intraclass correlation coefficent** |
| --- | --- | --- | --- | --- | --- | --- | --- |
| Training planning endurance | .63 - .88 | .39 - .77 | 0.96 | 0.60 | .60 | .59 (TPS) | .74 |
| Training planning strength | .71 - .89 | .51 - .80 | 0.98 | 0.72 | .72 | .65 (TES) | .83 |
| Training execution endurance | .65 - .82 | .43 - .67 | 0.90 | 0.57 | .58 | .47 (TPE) | .76 |
| Training execution strength | .66 - .86 | .43 - .74 | 0.91 | 0.56 | .58 | .65 (TPS) | .85 |
| Training monitoring and adjustment endurance | .72 - .85 | .52 - .73 | 0.92 | 0.63 | .63 | .59 (TMS) | .81 |
| Training monitoring and adjustment strength | .74 - .88 | .55 - .78 | 0.95 | 0.72 | .72 | .62 (TPS) | .85 |

Table 9. Results of measurement invariance testing across groups (sex, sport activity, and sports club membership).

|  | **SB-χ²** | **df** | **P** | **CFI** | **RMSEA** | **LO90** | **HI90** | **SRMR** | **ΔCFI** | **ΔRMSEA** | **ΔSRMR** |
| --- | --- | --- | --- | --- | --- | --- | --- | --- | --- | --- | --- |
| **Sex (binary)** | | | | | | | | | | | |
| Configural invariance | 6926.929 | 3508 | .000 | 0.925 | 0.049 | 0.047 | 0.050 | 0.045 | - | - | - |
| Metric invariance | 6991.851 | 3563 | .000 | 0.925 | 0.048 | 0.047 | 0.050 | 0.048 | 0.000 | 0.001 | 0.003 |
| Scalar invariance | 7162.275 | 3618 | .000 | 0.922 | 0.049 | 0.047 | 0.050 | 0.048 | 0.003 | 0.001 | 0.000 |
| **Sport activity (yes-no)** | | | | | | | | | | | |
| Configural invariance | 7064.212 | 3508 | .000 | 0.917 | 0.049 | 0.048 | 0.051 | 0.046 | - | - | - |
| Metric invariance | 7165.050 | 3563 | .000 | 0.916 | 0.049 | 0.048 | 0.051 | 0.051 | 0.001 | 0.000 | 0.003 |
| Scalar invariance | 7253.421 | 3618 | .000 | 0.916 | 0.049 | 0.047 | 0.051 | 0.050 | 0.000 | 0.000 | 0.001 |
| **Sports club membership (yes-no)** | | | | | | | | | | | |
| Configural invariance | 6943.137 | 3508 | .000 | 0.922 | 0.049 | 0.047 | 0.051 | 0.046 | - | - | - |
| Metric invariance | 7014.347 | 3563 | .000 | 0.922 | 0.048 | 0.047 | 0.050 | 0.050 | 0.000 | 0.001 | 0.004 |
| Scalar invariance | 7109.359 | 3618 | .000 | 0.922 | 0.048 | 0.047 | 0.050 | 0.050 | 0.000 | 0.000 | 0.000 |

Notes: SB-χ² = Satorra–Bentler scaled chi-square; df = degrees of freedom; p = significance level; CFI = comparative fit index; RMSEA = root mean square error of approximation; LO90/HI90 = lower and upper bounds of the 90% confidence interval of RMSEA; SRMR = standardized root mean square residual; ΔCFI, ΔRMSEA, and ΔSRMR indicate changes in model fit between nested models (configural, metric, scalar).
